# The Treatment of Syphilis

**Published:** 1892-01

**Authors:** 


### Selections and Abstracts

### THE TREATMENT OF SYPHILIS.

Hefte 8 u. 9. 1891, of the *Wiener Klinik* contain the valuable work of Dr. Elsenberg on the treatment of syphilis. After discussing the curability of this disease, the question of re-infection, the results of the excision of the primary lesion, he comes to a consideration of the means at our disposal for the successful treatment. For the primary lesion, if phagedenic, the ordinary antiseptics are recommended: corrosive sublimate, carbolic acid, iodoform, derivaties of iodine. He recommends the method of inunction as the best of all for external use of mercury. He insists that a full warm bath which contains a pound of soda, the patient being well rubbed with soap, shall be taken for two or three days before treatment; that the teeth shall be put in order, and any inflammation of the mucous membrane of the mouth shall be treated by washes of 5 per cent. solution of salol dissolved in alcohol and well diluted with water. For inunction he advises the ordinary blue ointment popularized by Lebeuf, in doses from one-half to one and a half drachms. The time required for thorough inunction of the minimum quantity is upward of half an hour. This treatment is to be continued until all symptoms have disappeared, or eight or ten more treatments have been made, or there intervenes swelling of the oral mucous membrane. For six or eight weeks succeeding this treatment the iodide of soda or potash is to be employed. Although placing his reliance upon the inunction method, yet the author gives a very complete history of the various mercurials used for internal medication, as well as those used hypodermatically, both the soluble and insoluble salts.

In the administration of the iodides he prefers the iodide of potash, in doses sufficient to accomplish his purpose, even to one hundred and fifty grains *per diem.* The iodide of soda is to be substituted when potash is contra-indicated, because of the condition of the heart. In rare cases iodoform or odol (thirty to forty-five grains *per diem*) may be substituted.

[Although but little that is new is to be found in this monograph, yet the litrature has been so thoroughly studied, the opinions of the clinicians discussed with so much fairness, the indications for treatment so clearly given, the methods to be employed so accurately described, that the careful perusal of this work will amply repay one who wishes to keep himself fully informed on the subject.—ED.]—*Amer. Jour. Med. Sciences.*

